# Dynamic Mechanical Properties of Coals Subject to the Low Temperature-Impact Load Coupling Effect

**DOI:** 10.1038/s41598-019-56755-7

**Published:** 2019-12-27

**Authors:** Yanbing Wang, Yang Yang, Yuantong Zhang, Jianguo Wang

**Affiliations:** 1 School of Mechanics and Architecture Engineering, China University of Mining and Technology (Beijing), Beijing, 100083 China; 2 College of Water Conservancy, Yunnan Agricultural University, Kunming, Yunnan 650201 China; 3 College of Civil and Architectural Engineering, Yunnan Agricultural University, Kunming, Yunnan 650201 China

**Keywords:** Environmental sciences, Solid Earth sciences

## Abstract

The effects of low temperature gradient on dynamic mechanical properties of coal were examined through Split-Hopkinson Pressure Bar (SHPB) Dynamic Impact Experiment, the law of dynamic mechanical parameters of coal specimen changing from room temperature to negative temperature (25 °C, −5 °C, −10 °C, −15 °C, −20 °C, −30 °C, −40 °C) was analyzed, and the coal-rock stress-strain curve characteristics subject to the low temperature-impact load coupling effect was discussed. The law of the effects of different strain rates on the rock strength properties and the compressive deformation and failure was analyzed by studying the coal specimen at −15 °C. Moreover, the mechanical properties of coal specimen in saturated and dry conditions were compared, and the effects of water and water-ice phase transition on the strength properties of the corresponding coal specimen were analyzed.

## Introduction

With the balanced development of Chinese regional economy and the deepening of “Great Western Development Strategy”, the coal mining quantity in high altitude, cold, frozen even glacier-deposited areas have been increasing. It will be of great significance to the resource exploitation and utilization in cold areas by studying the hydrological, mechanical and dynamic mechanical properties of the low temperature coal-rock in these special geological environments. In addition, the research into the dynamic mechanical properties of the low temperature coal-rock subject to impact, explosion and other dynamic loads will be referential to the planning, design and construction of mine construction project in coal areas.

Recently the rock mechanical properties at low temperature have been studied, but very few studies observe the coal. Y. Inada and K. Yokata^1^ made statistics of the rock strength characteristics at −160 °C, results show that the uniaxial compressive strength and the tensile strength of rock are not sensitive to the moisture content; they would increase as the temperature fell, which is attributed to the shrinkage of rock mineral particles and the increase of pore ice strength at low temperature. K. Aoki *et al*.^2^ tested the mechanical and thermal properties of various rocks within the low temperature ranges, and results show that compressive strength, tensile strength and elastic modulus markedly increased at −160 °C. T. Yamabe and K. M. Neaupane^3^ tested the thermodynamic properties of sandstone at low temperature through uniaxial and triaxial experiment, and fit and characterized the related law, in which the tensile and compressive strength of rock would increase as the temperature fell, but in −10 °C–20 °C, although the compressive strength was increasing, the elastic modulus basically didn’t change and was not affected by temperature. R. D. Dwivedi^4^ tested the fracture toughness of eight rocks at low temperature using Brazilian disc experiment, found that the rock fracture toughness at low temperature was significantly higher than that at room temperature, and tended to increase linearly as the temperature fell. D. Nakamura^5^ considered that the temperature and position at which the ice lens in the rock was formed are irrelevant to the temperature gradient, but solely depends on the internal rock composition and its appropriate physical properties, through low-temperature freezing experiment of sandstone and tuff. N. Matsuoka^6^ carried out a lot of freezing-thawing failure experiments on various rocks, and asserted that the rock freezing-thawing damage and failure is primarily attributed to the frost heaving effect of capillarity attraction and pore ice by semi-immersing and thawing the frozen rock in the water. M. Mutlutuk *et al*.^7^ through repeated freezing-thawing cycle experiment found that the temperature change would damage the rock integrity, more freezing-thawing cycles and higher frequency will lead to greater loss on the rock integrity and more obvious damage and failure characteristics. T. C. Chen *et al*.^8^ in a tuff freezing-thawing cycle experiment found that the rock damage and deterioration has a critical saturation rate, when the tuff saturation rate was lower than 60%, the damage and deterioration in the freezing-thawing cycle would be not obvious, but when the saturation rate was higher than 70%, the repeated freezing-thawing cycle would quickly lead to rock failure. K. Watanabe^9^ studied the law of the effects of moisture content and supercooled water on the freezing-thawing rock strength and deformation characteristics, based on the freezing-thawing cycle experiment of the rocks with different moisture rates. K. Hall^10,11^, J. P. McGreevy and W. B. Whalley considered that the initial moisture rate was the essential cause to the damage and failure of freezing-thawing rock, and proved that the saturated rock may lead to the rapid crack propagation subject to the freezing effect, through the freezing experiment on the bedrock in water-rich area. M. Fukuda^12^ and J. P. Lautridou^13^ studied the effects of rock pore rate on the damage and deterioration of freezing-thawing rock, and considered that the rock with a pore rate of more than 20% is likely to be affected by frost heaving force, the damage and failure basically appeared after 30 freezing-thawing cycles, while the rock with a pore rate of less than 6% basically does not change even though after hundreds of freezing-thawing cycles. Looking at the above-mentioned documents, it can be found that their research material is almost rock. The rock and the coal still have a certain difference, the strength of the rock is high, the water holding capacity is weak, and the coal is the result of the result, the soft, the porosity is large. And the mineral composition of the coal also has a great difference with the rock. The above-mentioned studies are mainly aimed at the mechanical properties of frozen rock under static load, and few of them are dynamic. In the static test, the physical and mechanical properties of the rock under low temperature have changed, the strength of the rock is increased with the decrease of the temperature, but after a certain temperature is reduced, the strength is no longer changed. What’s more, they believe that both temperature and the number of freeze-thaw cycles will have an impact on the mechanical properties of rock.

In this work, the effects of low temperature gradient on the dynamic mechanical properties of coal were studied through SHPB Dynamic Impact Test, the law of dynamic mechanical parameters of coal specimen changing from room temperature to negative temperature was analyzed, the mechanical properties of coal specimen in saturated and dry conditions were compared, and the effects of water and water-ice phase transition on the strength properties of the corresponding coal specimen were analyzed. The law of the effects of different strain rates on the rock strength properties and the compressive deformation and failure was analyzed by studying the coal specimen at −15 °C.

## Experiment Details

### Preparation of coal specimens

**Figure 1:**
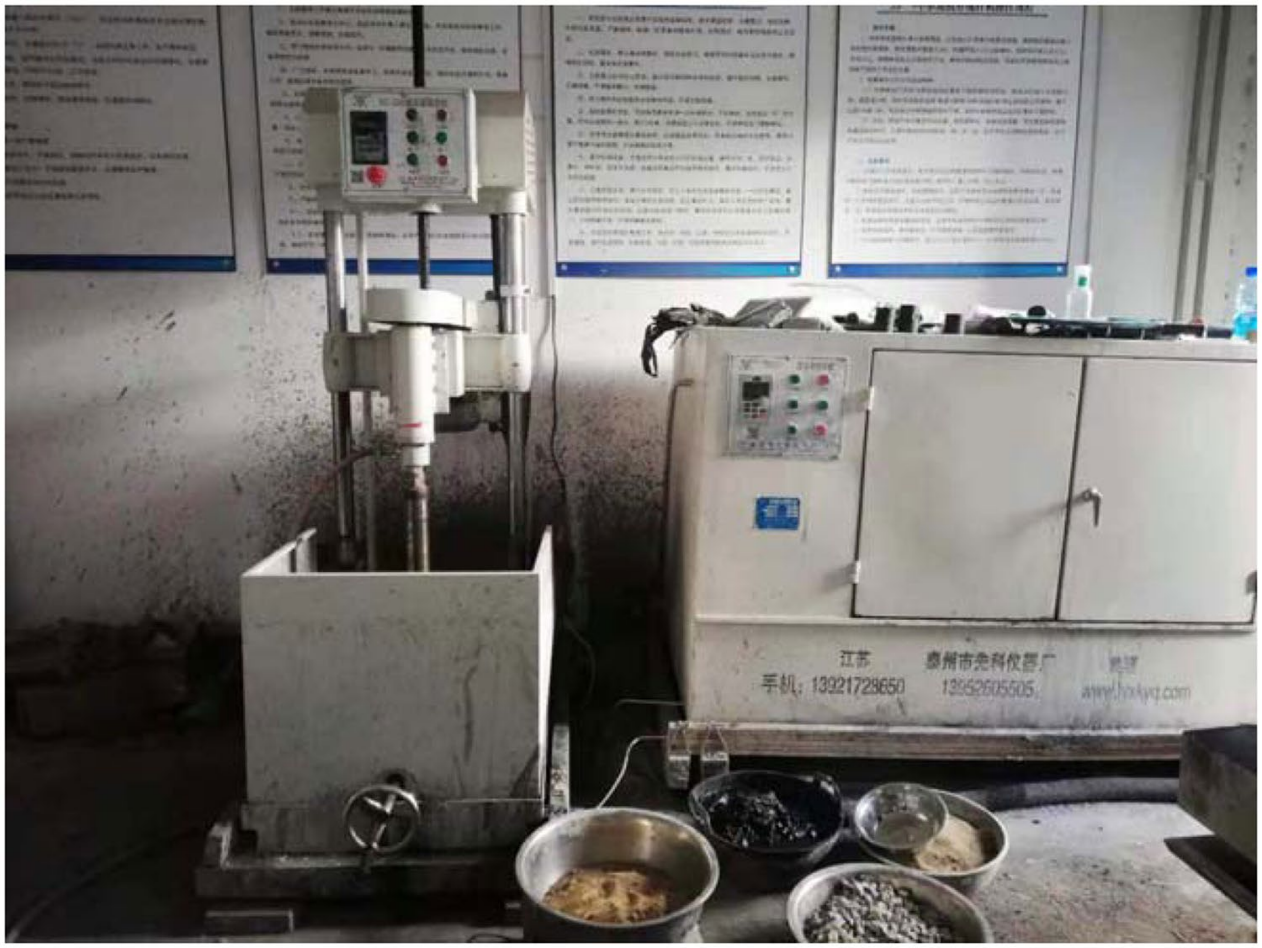
Specimen processing unit.

**Figure 2:**
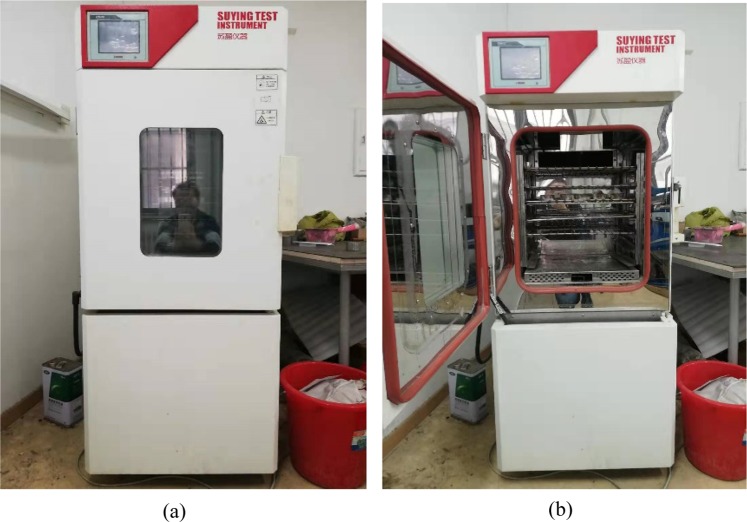
HT-LT test instrument.

Coal specimens were sourced from The Second Mining Area, Shuguang Mine of Shanxi Coking Coal. Before the experiment, the air dried moisture, ash yield and volatile matter yield of the coal were examined. The average value of them were 7.35%, 10.2%, and 28.7% respectively. The main mineral components of coal samples are quartz, pyrite, kaolinite, Illite, magnetite, gypsum and calcite. Through laboratory chemical analysis, the content of inorganic minerals in the samples is 16.08%. Among them, quartz is 10.36%, clay mineral is 5.78%, pyrite is 0.43%, carbonate mineral is 0.59%.To ensure a certain relevance of the physical and mechanical properties of the specimens, all specimens were cut and processed from a complete coal-rock sample, each specimen has dimensions of Φ50 mm × 25 mm, average diameter of 49.45 mm, average thickness of 25.18 mm, with a size error of ±1 mm, the surface unevenness after grinding two end faces is ±0.05 mm, each end face is perpendicular to the axis, with the maximum deviation of no greater than 0.25°. Figure 1 shows a special unit for processing specimens. The ground specimens were saturated, then coated with Vaseline and wrapped with preservative film, placed in HT-LT test instrument for freezing. Figure 2 shows the HT-LT test instrument produced by Shanghai Suying and the frozen specimens. To properly explain this, a few coal specimens were dried for comparison. In particular, when the sample is saturated with water, the mass is increased by about 4.3% to 6.1%.

Two kinds of water-bearing coal samples, dry and saturated, will be used in the experiment. The preparation methods of dry coal samples are as follows: put the selected samples into the oven, bake at 105  °C for 48 hours to constant weight (the mass change does not exceed 0.1% within 24 hours), and then weigh and record the quality of each rock sample; The preparation method of saturated coal sample is as follows: the drying specimen is placed in a vacuum water satiator, the air in the container is extracted by 0.1 MPa pressure, distilled water is injected into the container after pumping for 2 hours, the water surface should be higher than that of the specimen, and the air pumping continues for 4 hours until there is no bubble overflow, and then the quality of the specimen is weighed and recorded after 48 hours under atmospheric pressure.

### Test unit

#### SHPB experiment system

**Figure 3:**
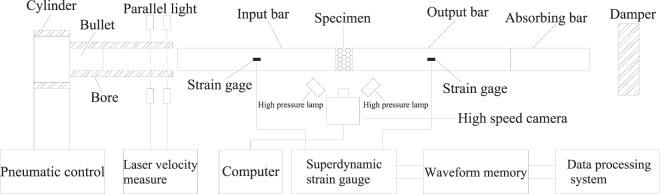
Diagram of SHPB experiment system.

The impact compression experiment was carried out on the Split-Hopkinson Pressure Bar (SHPB) experimental system from State Key Laboratory for Geomechanics & Deep Underground Engineering, China University of Mining & Technology, Beijing, as shown in Fig. 3. In this experiment, the diameter of steel cylindrical bullet, input bar and output bar of SHPB unit is 50 mm, with a length of 400 mm, 2,000 mm and 2,000 mm respectively, the strain gages were attached on the input bar and output bar at 1 m from the end of specimen, so as to record the strain of bar. The initial velocity of bullet was controlled by the air pressure in the chamber, while the velocity of input bar was measured using photoelectric method. This experiment was based on the following assumptions: (1) One-dimensional stress wave. Assume the specimen and press bar meet the unaxial stress state in the course of the experiment, the stress, displacement and strain at the specimen-press bar interface can be solved according to the basic theories of one-dimensional stress wave; (2) Uniformity, assume the stress and strain of the shorter specimen are uniformly distributed along its length, and it can stretch radially, the radial, axial inertial effects and the friction effects of the end face are neglected^14,15^.

#### Test method

According to the assumption of stress uniformity, the dynamic stress-strain relation of the material can be obtained using three-wave method^14^:

1 $$\varepsilon (t)=\frac{c}{{l}_{s}}{\int }_{0}^{t}({\varepsilon }_{i}-{\varepsilon }_{r}-{\varepsilon }_{t}){\rm{d}}t$$

2 $$\sigma (t)=\frac{A}{2{A}_{s}}E({\varepsilon }_{i}+{\varepsilon }_{r}+{\varepsilon }_{t})$$

3 $$\dot{\varepsilon }(t)=\frac{c}{{l}_{s}}({\varepsilon }_{i}-{\varepsilon }_{r}-{\varepsilon }_{t})$$

where, *E*, *c*, *A* denotes the elastic modulus, elastic wave velocity and cross-sectional area, respectively; *A*_*s*_, *l*_*s*_ denotes the initial cross-sectional area and the initial length of specimen, respectively; *ε*_*i*_, *ε*_r_, *ε*_t_ denotes the incident, reflection and transmission strain in the bar.

The purpose of this experiment is to fully examine the dynamic compression performance of coal subject to the low temperature-impact load coupling effect, which was carried out in respect of the temperature effect and the strain rate effect and can be divided into two parts:

1. Temperature effect experiment of mechanical properties of the coal specimen at high strain rate.
  In the experiment, the coal specimens were saturated (a few were dried), then placed in the HT-LT test instrument, the temperature was decreased at a constant rate (−5 °C/h) to −5 °C, −10 °C, −15 °C, −20 °C, −30 °C, −40 °C respectively, and used for the impact temperature at a high strain rate after being kept at such temperature for 24 h. With the room temperature (25 °C) experiment group, this experiment can be divided by temperature gradient into 7 groups, each group at least has 3 specimens, the specimens after low-temperature treatment were transferred quickly to the SHPB Test Unit with a diameter of Φ50 mm, the uniaxial impact compression test of coal specimens at a low temperature was carried out by applying 0.50 MPa impact pressure.
2. Strain rate effect experiment of dynamic mechanical properties of the coal specimen at low temperature.

−15 °C was selected as the freezing temperature for coal specimens. In the experiment, the saturated coal specimens were decreased at a constant rate (−5 °C/h) to −15 °C and kept at such temperature for at least 24 h, then applied with the impact pressure of 0.50 MPa, 0.55 MPa, 0.60 MPa, 0.65 MPa respectively, so as to study the coal deformation properties, mechanical parameters and macro failure characteristics subject to the uniaxial compression conditions, and analyze the strain rate effect of dynamic mechanical properties of coal specimens at a low temperature.

## Temperature Effect Experiment of Dynamic Mechanical Properties of the Coal Specimen at High Strain Rate

This experiment intended to examine the temperature effect of dynamic mechanical properties of coal specimen at a high strain rate changing from room temperature to low temperature, given the same axial impact load and using temperature as the variable. In consideration of high absorption rate of coal, the effects of water on the dynamic mechanical properties of coal specimen, especially the low-temperature frozen specimen were analyzed with reference to the dry control group.

### Stress-strain curve

**Figure 4:**
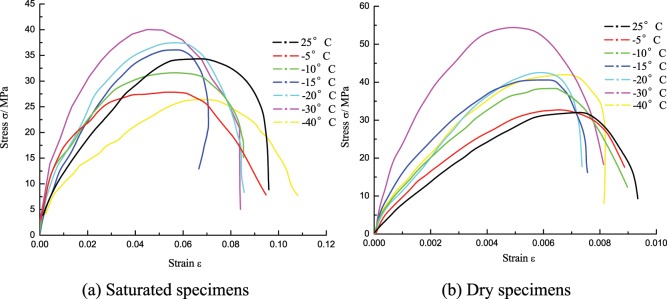
Stress-strain curve of coal specimens at different temperatures.

In the experiment, the same loading pressure (at 0.50 MPa) was controlled to achieve similar impact velocities, and the stress-strain curve for saturated specimens and dry specimens at the corresponding temperature was obtained through the impact experiment at different low temperatures. From Fig. 4, they showed the same law of change and went through four stages: densification, elastic deformation, yield and failure.

### Effects of low temperature gradient on the strength performance of coal specimens

The experimental data was processed through “three-wave method” to acquire the peak strength of the corresponding coal specimen at different temperatures, see Tables 1, 2.

**Table 1:** Peak strength of the saturated specimen at different temperatures.

Temperature/°C	Specimen No.	Loading Pressure/MPa	Impact Velocity/m·s^−1^	Peak Strength/MPa	Average Value/MPa
25	SC-1	0.50	3.75	28.08	29.50
	SC-2		3.74	33.68	
	SC-3		3.77	26.75	
−5	SC-4	0.50	3.77	33.35	34.16
	SC-5		3.77		
	SC-6		3.78	40.77	
	SC-7		3.76	25.14	
−10	SC-8	0.50	3.77	42.10	37.72
	SC-9		3.79	38.35	
	SC-10		3.75	32.71	
−15	SC-11	0.50	3.74	40.02	38.38
	SC-12		3.74	36.78	
	SC-13		3.77	38.33	
−20	SC-14	0.50	3.74	41.44	40.34
	SC-15		3.74	39.22	
	SC-16		3.77	40.35	
−30	SC-17	0.50	3.79	42.74	42.71
	SC-18		3.79	45.39	
	SC-19		3.80	38.99	
−40	SC-20	0.50	3.76	35.48	35.43
	SC-21		3.79	36.44	
	SC-22		3.75	34.39

**Table 2:** Peak strength of the dry specimen at different temperatures.

Temperature/°C	Specimen No.	Loading Pressure/MPa	Impact Velocity/m·s^−1^	Peak Strength/MPa	Average Value/MPa
25	CD-1	0.50	3.74	32.04	32.50
	CD-2		3.75	31.89	
	CD-3		3.78	33.57	
−5	CD-4	0.50	3.73	44.44	38.17
	CD-5		3.75	31.68	
	CD-6		3.75	38.38	
−10	CD-7	0.50	3.70	35.69	39.11
	CD-8		3.71	44.11	
	CD-9		3.77	37.52	
−15	CD-10	0.50	3.75	29.00	39.59
	CD-11		3.70	48.36	
	CD-12		3.77	42.51	
−20	CD-13	0.50	3.73	29.10	40.00
	CD-14		3.75	48.56	
	CD-15		3.76	42.35	
−30	CD-16	0.50	3.70	30.74	45.82
	CD-17		3.76	53.05	
	CD-18		3.77	53.65	
−40	CD-19	0.50	3.77	54.48	44.66
	CD-20		3.78	45.44	
	CD-21		3.78	34.06	

**Figure 5:**
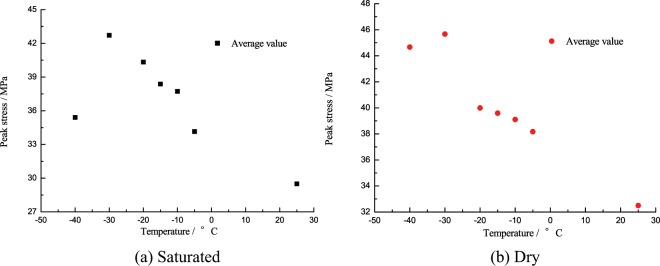
Peak strength of saturated specimen changing with temperature.

Shan Renliang^16^ through the static load compression test of saturated red sandstone at negative temperature found that the peak strength of red sandstone will increase exponentially as the temperature falls (10 °C~−15 °C). However, in this dynamic compression experiment, the tendency of the strength performance of saturated specimens changing with temperature is markedly different from that of static loading experiment, as shown in Fig. 5(a), when the temperature fell from 25 °C to −5 °C, the peak strength of saturated specimens changed from 29.50 MPa to 34.16 MPa, up by 4.1%; in −5 °C~−30 °C, the peak strength of saturated specimens tended to increase as the temperature fell, reached 37.72 MPa, 38.37 MPa, 40.34 MPa, 30.53 MPa at −10 °C, −15 °C–20 °C, and −30 °C respectively, up by 14.9%, 16.9%, 22.9%, 24.0% if compared to −5 °C; when the temperature fell from −30 °C to −40 °C, the peak strength of saturated specimens decreased significantly to 35.43 MPa from 42.71 MPa at −30 °C, down by approximately 42.2%.

**Figure 6:**
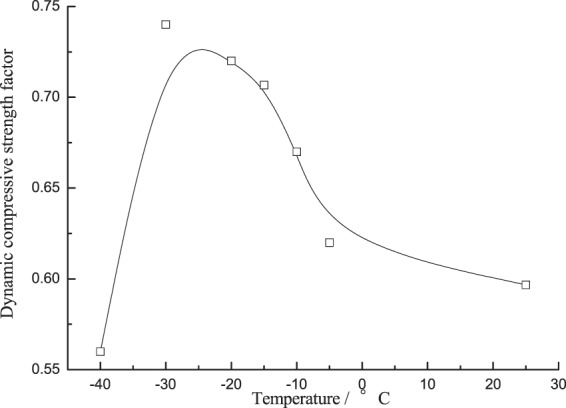
Dynamic compressive strength factor changing with temperature.

The dynamic compressive strength factor η is the ratio of dynamic compressive strength to static compressive strength, which reflects the change of response characteristics of coal-rock material subject to the dynamic loading, as mainly indicated by the increase of failure strength. The law of changes of dynamic compressive strength factor is consistent with the corresponding changes of strength, if compared with the data in ref. ^17^, as shown in Fig. 6, it was found that the η of saturated specimens would gradually increase then drastically decrease as the temperature fell, in which the dynamic strength factor plunged in −30 °C~−40 °C, down by 30%.

Low temperature is a critical factor impacting the strength performance of coal-rock in the dynamic mechanical experiment, but its way and effect of impacting is very different from that in the static loading test. Prior studies of static loading strength characteristics^16^ reported that the negative temperature coal-rock at athe uniaxial and triaxial state tends to increase as the temperature falls, which has reached a general agreement. However, the strength characteristics of coal specimens in the dynamic mechanical experiment become more complex due to presence of water and water-ice phase transition. But in general, the presence of water in the coal-rock will aggravate the impact of temperature decrease on the coal-rock dynamic mechanical properties.

### Effects of low temperature gradient on the peak strain of coal specimens

**Figure 7:**
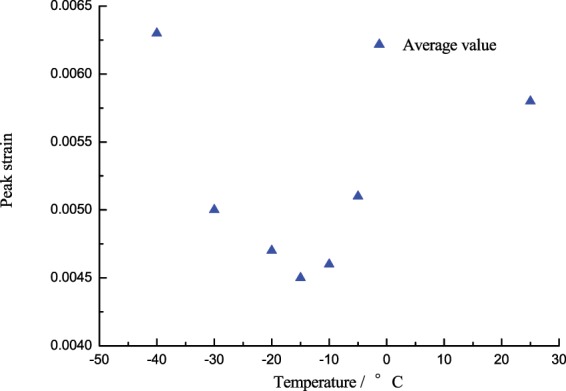
Peak strain of the coal specimen changing with temperature.

Peak strain is a critical indicator used to measure the coal-rock deformation characteristics subject to the ultimate load. The study of peak strain at a high strain rate would determine the change characteristics of the coal-rock in a specific condition, analyze its ductile-brittle characteristics, and provide appropriate data support for the real rock breaking project. The law of the peak strain of the corresponding saturated specimen changing with temperature will be obtained by analyzing the stress-strain curve of saturated specimen at different temperatures and high strain rates. Table 3 shows the law of the peak strain of the saturated specimen changing with temperature. And Fig. 7 is plotted according to the available data in Table 3.

**Table 3:** Peak strain of the coal specimen changing with temperature.

Peak strain	*T*/°C						
	25	−5	−10	−15	−20	−30	−40
Single specimen	0.0059	0.0050	0.0048	0.0046	0.0048	0.0050	0.0057
	0.0055	0.0051	0.0046	0.0045	0.0047	0.0049	0.0065
	0.0059	0.0051	0.0045	0.0044	0.0046	0.0049	0.0067
Average value	0.0058	0.0051	0.0046	0.0045	0.0047	0.0050	0.0063

From Fig. 7, the law of the peak strain changing with temperature is markedly different from that of the peak stress. At the turn point of −10 °C, the peak strain of the saturated specimen will decrease as the temperature fell before −10 °C (25 °C~−10 °C), while gradually increase as the temperature fell after −10 °C (−10 °C~−40 °C).

1. In 25 °C~−10 °C, the peak strain continuously decreased from 0.0055 at 25 °C to 0.0046 at −5 °C, until it reached 0.0043, down by 22.0%, in which the decrease of strain in −5 °C~−10 °C was greater than that in 25 °C~−5 °C. As the temperature fell, the mineral particles shrunk, the coal specimen tended to transform from ductile-brittle to brittle performance, the anti-deformation capacity enhanced, so that the peak strain tended to decrease, but 0 °C~−5 °C is a special range where the water-ice phase transition was present, the water-ice phase transition volume expanded by 9%. For saturated specimens, the expansion of ice volume led to the generation of frost heaving force, aggravated the internal damage of the specimen, and enhanced the deformability, so that the strain decrease in 25 °C~−5 °C was slightly small.
2. In −10 °C~−40 °C, the peak strain of saturated specimens would increase as the temperature fell, the peak strain at −20 °C, −30 °C, −40 °C was 0.0047, 0.0050, and 0.0063 respectively, up by 10.2%, 13.3%, 47.7% respectively compared to −10 °C. After −10 °C, as the temperature fell, the coal matrix and the ice volume will shrink as the temperature fell, but the shrinkage rate of ice was greater than that of the coal matrix^18^, different shrinkage rates of these two media resulted in new defects, pores and cracks in the specimen, the mechanical performance deteriorated; subject to the impact load, the bearing capacity reduced, the deformation aggravated, and the deterioration got worse and the peak strain continuously increased as the temperature fell, which is indicated in macro sense by the increase of fragments, the reduction of volume and the serious breaking after failure.

## Strain Rate Effect of the Dynamic Mechanical Properties of the Coal Specimen at Low Temperature

Considering the temperature in cold and freezing areas of China and the low temperature technology used in the freezing construction, this section observed the saturated specimens at −15 °C to analyze the law of its mechanical properties changing at different strain rates.

### Change characteristics of the stress-strain curve

**Figure 8:**
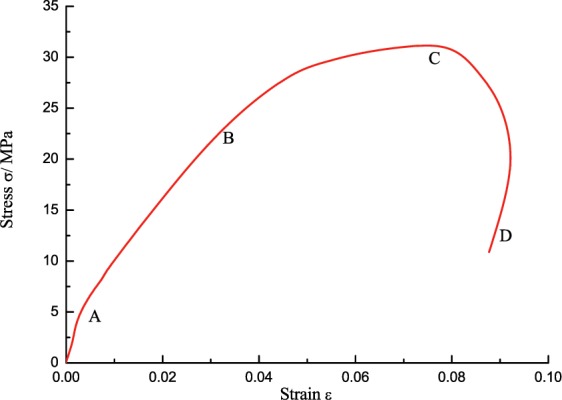
Dynamic stress-strain curve of the saturated specimen at −15 °C.

**Figure 9:**
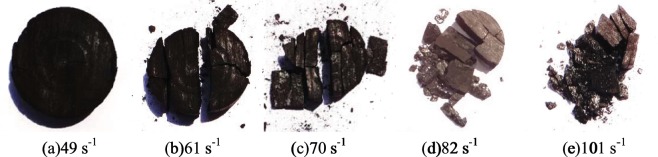
Failure form at different strain rates.

As shown in Fig. 8, the stress-strain curve of low-temperature coal specimens can be also divided into four stages, each stage has its appropriate characteristics: ① The linear elastic segment with relatively large slope (OA), compared to the room temperature, the proportion of the linear elastic segment in the whole curve increased and the slope sharply increased, which indicates that the initial modulus of the coal-rock at low temperature increased and its anti-deformation capacity enhanced. ② The longer elastic deformation segment (AB), during which the elastic modulus significantly decreased. Compared to the non-linear deformation of AB at room temperature, this curve segment at low temperature was closer to a linear elastic change, its length was 45% of the whole curve, with obvious brittle characteristics. In this stage, the stress increased steadily with the strain, the internal microcracks initiated and a few propagated stably, the linear elastic deformation predominated the coal-rock deformation. ③ The shorter crack propagation segment (BC), a lot of internal microcracks initiated and transited from stable propagation to unstable propagation, the primary cracks quickly grew to cut through the coal specimen, until a micro failure happened at Point C of the specimen, the stress reached the peak. ④ The quick unloading segment (CD), the stress-strain curve quickly decreased at a certain rate after experiencing a transient course of strain softening, which indicates the stronger coal-rock brittle performance, and further confirms the rock transformed from ductile-brittle to brittle characteristics at low temperature. Figure 9 shows the failure form of the coal specimen at different strain rates.

### Effects of strain rate on the stress-strain curve characteristics

**Figure 10:**
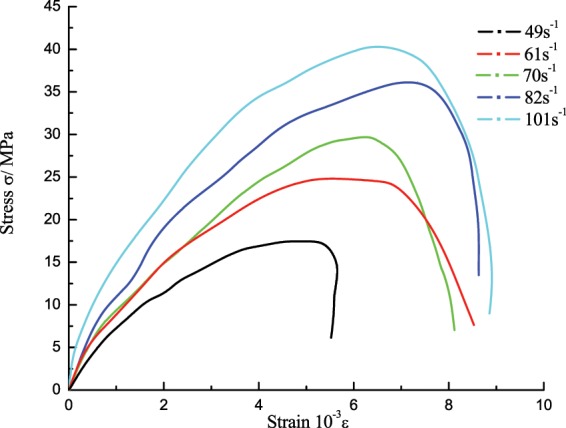
Stress-strain curve of the coal specimens at different strain rates.

Figure 10 shows the stress-strain curve of the coal specimens at different strain rates, the waveform curves at low temperature showed various trends, but as the strain rate changed, some common characteristics have the following trends: ① As the strain rate increased, the proportion of the elastic deformation stage of the coal specimen in the pre-peak stress-strain curve increased, which indicates that the higher strain rate means the stronger elastic deformation behavior of the coal-rock, and the higher energy input acquired from the specimen per unit time, so that the specimen will store a lot of elastic deformation energy to facilitate the initiation and propagation of microcracks. ② The strain length of the specimen during strain softening will gradually increase as the strain rate rose, because more cracks will be involved in the deformation process of the specimen as the strain rate increased, its post-peak bearing capacity will rapidly decrease and the softening deformation will increase. ③ As the strain rate increased, the dynamic elastic modulus and the peak stress of the specimen will increase, which shows the same law of changing as that at room temperature.

### Law of peak stress changing with the strain rate

**Figure 11:**
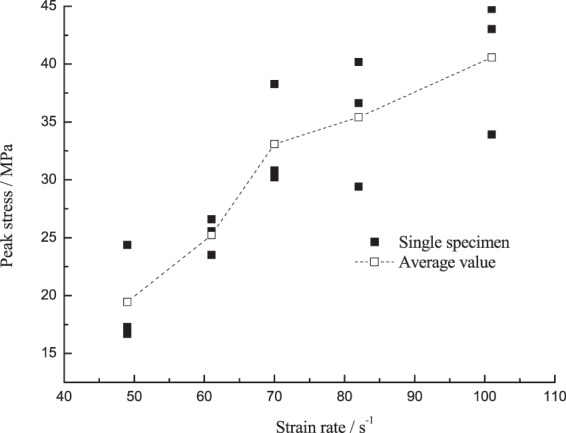
Peak stress of the coal specimen changing with the strain rate.

**Figure 12:**
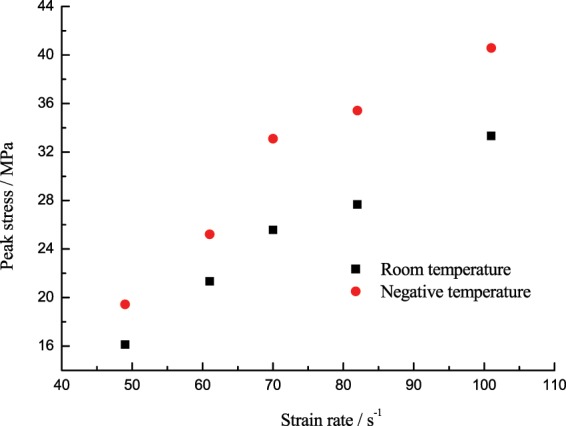
Comparison of average values of the peak stress at negative temperature and room temperature.

The experimental data in Fig. 11 plotted the peak stress of the corresponding specimen changing with the strain rate at −15 °C. Figure 12 is the comparison curve of average values of the peak stress at negative temperature and room temperature. From Fig. 12, the peak stress of the specimen will gradually increase as the strain rate increased at room temperature and −15 °C; given the same range of strain rate, the peak stress at −15 °C was greater than that at room temperature, because the strength increase caused by the shrinkage of the coal-rock matrix and the ice medium at low temperature was greater than the strength deterioration resulting from the water-ice phase transition. Such results correspond to the temperature effect test results at high strain rates as stated above.

The peak stress of the low-temperature frozen coal specimen subject to the impact load changing with the strain rate can be divided into two stages: (1) The strain rate increased from 49 s^−1^ to 70 s^−1^, the peak stress of the specimen rapidly increased from 19.45 MPa to 24.82 MPa, up by 70.2%; (2) The strain rate increased from 70 s^−1^ to 101 s^−1^, the peak stress of the specimen slowly increased from 24.82 MPa to 40.57 MPa, up by 22.56%. It was found through comparison of the peak stress increase that the ultimate bearing capacity of the specimen gradually increased as the strain rate increased at low temperature, but its increase slowed down at higher strain rates.

### Law of dynamic elastic modulus changing with the strain rate

**Figure 13:**
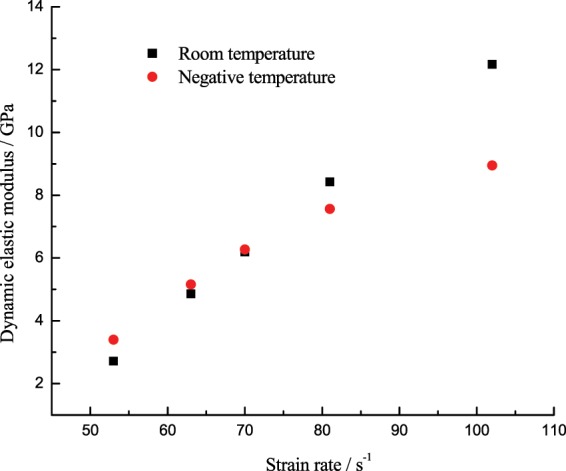
Dynamic elastic modulus of the coal specimen changing with the strain rate.

“Elastic modulus” was firstly defined as the corresponding slope of 40~60% of the compressive strength in the rising segment of the stress-strain curve, and the equation is described as follows:

4 $${E}_{C}=\frac{{S}_{2}-{S}_{1}}{{\varepsilon }_{2}-{\varepsilon }_{1}}$$

where the subscript “1” and “2” denotes 0.4*σ*_0_, 0.6*σ*_0_ in the stress-strain curve, respectively; *S* means the axial stress; *ε* means the axial strain; the value of elastic modulus can be calculated from the stress-strain curve. Figure 13 shows the curve of elastic modulus changing with the temperature.

From Fig. 13, the dynamic elastic modulus of the specimen at room temperature and −15 °C will increase with the strain rate. At low strain rate, the dynamic elastic modulus of the specimen at −15 °C was greater than that at room temperature, but as the strain rate increased, the increase rate of dynamic elastic modulus at room temperature was quicker, until it surpassed the dynamic elastic modulus at −15 °C.

After the low-temperature freezing at −15 °C, when the strain rate increased from 49 s^−1^ to 101 s^−1^, the dynamic elastic modulus of the saturated specimen increased by 4 times, in 49 s^−1^–70 s^−1^, the dynamic elastic modulus increased quickly from 3.40 GPa to 6.27 GPa, up by 84.6%; then its growth slowed down, increasing from 7.57 GPa at 82 s^−1^ to 8.95 GPa at 101 s^−1^, up by only 18.3%.

### Peak strain changing with the strain rate

**Figure 14:**
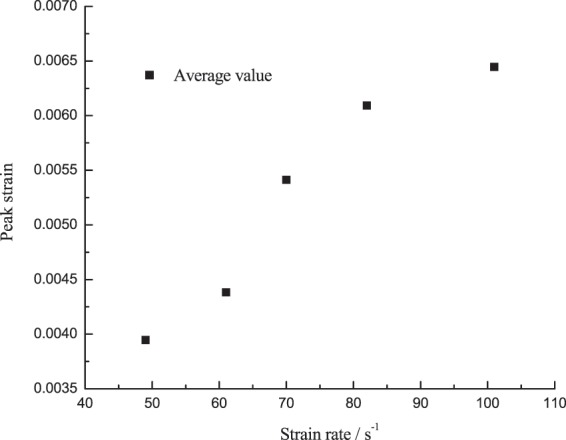
Curve of peak strain of the specimen changing with the strain rate.

Figure 14 shows the peak strain of the corresponding specimen changing with the strain rate at low temperature, which suggests that the peak strain of the specimen at −15 °C will increase with the strain rate, and the change of the specimen can be obviously divided into two stages: (1) The strain rate increased from 49 s^−1^ to 70 s^−1^, the peak strain quickly increased from 0.0039 to 0.0054, up by 37.2%; (2) The strain rate increased from 70 s^−1^ to 101 s^−1^, its growth slowed down, increasing from 0.0054 to 0.0064, up by 19.1%.

## Discussion: Effects of Water-ice Phase Transition on the Coal-rock Dynamic Mechanical Properties

From Fig. 5(a,b), the tendency of peak strength of saturated specimen and dry specimen changing with the temperature is somewhat different. The strength of the dry specimen will increase then decrease as the temperature fell, the turning point of temperature was −30 °C; similarly, the strength of the saturated specimen increased then decreased at the turning point of −30 °C, but the strength increase in 25 °C~−5 °C was as little as 4.0%, such increase in dry state was 14.1%. Except the overall strength increase of the specimen at low temperature, the water-ice phase transition will weaken the mechanical properties of saturated specimen strength to some extent; after −30 °C, the specimen began to deteriorate subject to the low temperature effect, the strength loss for saturated specimen and dry specimen was 30.0%, 4.7% respectively, the strength of the saturated specimen was seriously damaged, which can be attributed to the sharp differences in shrinkage rate between the ice medium and the coal matrix at negative temperature.

With reference to^16,19^, most studies on the low-temperature frozen coal-rock materials focus on the static and quasi-static uniaxial or triaxial test, but almost no study observed the basic parameters of dynamic mechanical performance. Two main conclusions were drawn from this work: ① The uniaxial and triaxial compressive strength of the low-temperature frozen rock would increase exponentially as the temperature fell; ② The strength of the low-temperature frozen rock didn’t change drastically before −45 °C, then gradually increased as the temperature fell, namely stayed the same then increased. It is clear that these two trends are quite different from that of the dynamic strength curve, in 0 °C~5 °C, the water-ice phase transition had a little effect on the trends of the uniaxial and triaxial strength test results, but significantly affected the dynamic mechanical test results subject to the strong impact load. In this case, the water-ice phase transition would result in microcracks in the coal-rock, the primary cracks will further develop; in the static and quasi-static test, these microcracks and cracks were closed subject to the external load, the internal damage due to the volumetric expansion of the ice-water phase transition cannot be fully reflected, but the temperature decrease and the shrinkage of mineral particles led to the densified coal mass and the increased strength. However, the closure of the internal cracks in the coal mass and the propagation of new cracks were almost simultaneous subject to the strong impact load or stress wave, when the strain rate reached a certain critical value, the velocity of crack initiation will be much higher than that of the crack closure, while new cracks were initiated due to stress concentration at microcracks, micro-defects and microscopic weak structures. In this case, the deterioration of internal damages, defects and initiated microcracks resulting from the volumetric expansion of ice-water phase transition on the coal-rock mecahncial properties was fully reflected. Through experiment, it was found that in 0 °C~−5 °C, the degree of deterioration of temperature decrease on the dynamic compressive strength of saturated specimen was higher than the strength increase due to the cold shrinking coal medium. In −5 °C~−30 °C, the coal matrix and the ice would shrink as the temperature fell, and the shrinkage rate of the coal matrix was slightly higher than that of the ice, the microcracks, micro-defects caused by water-ice phase transition will close or disappear with the shrinkage of mineral particles; in this stage, the strength of coal specimen will increase as the temperature fell. After −30 °C, the shrinkage rate of the coal matrix was significantly higher than that of the ice medium. Furthermore, since the coal mass is composed of multiple minerals, the differences in shrinkage rate of multiple mineral particles at a low temperature (−30 °C) will be also fully amplified. Due to the sharp differences in shrinkage rate of multiple media which comprise the frozen coal specimen, cracks, defects and weak structures may further initiate on the structural plane of coal specimen and at the interface between the matrix and the ice, and the strength properties will be further deteriorated.

It is clear that the pore water had a critical effect on the coal-rock dynamic mechanical properties. Firstly, the volume of the part where the ice-water phase transition occurred will expand by about 9%, which was the source of the frost heaving force. The study of the frozen coal-rock damage and failure should focus on the basic feature of water-ice phase transition. Secondly, some water in the pore of medium which is even below the freezing point may keep the liquid state without freezing^20^. Based on these two points, the coal mass generally experienced a process of squeezing water due to the increase of frost heaving force in the early stage of the freezing, if there is enough free space in the crack medium to provide the squeezed free water, but the cooling capacity is not sufficient to make a large volume of water frozen, resulting in large enough macro frost heaving deformation, then the crack medium will be represented as freezing shrinkage. Otherwise, when the crack medium reaches a large saturation, there is no enough free crack space to provide the free water squeezed in the freezing process, the expansion of ice will predominate; in this case, the medium framework will expand and deform subject to the frost heaving force, which will be represented as frost heaving. Therefore, the freezing shrinkage should meet two conditions: lower saturation and higher freezing temperature; as long as the coal mass reaches a certain saturation, the coal mass below the freezing point will be represented as frost heaving as the freezing temperature falls, the coal specimen where the frost heaving happens will further occur freezing shrinkage as the temperature continuously falls, also referred to as “critical temperature of freezing shrinkage for the saturated specimen”. In this thesis, since the critical temperature of freezing shrinkage was in −5 °C~−10 °C, the saturated specimen will generally have frost heaving before freezing shrinkage within the range of negative temperature.

In this work, the surface of saturated coal specimens was coated with Vaseline and wrapped with preservative film prior to the freezing. It can be considered to be frozen at low temperature in a closed system. There was no migration of external moisture in the closed system, the frost heaving damage and failure occurring on the specimen can be primarily attributed to the frozen expansion of *in-situ* pore water. In addition, some water in the microcracks of the coal (namely the unfrozen water below the freezing point as mentioned earlier) will not be frozen due to the interfacial effect, it may migrate to the macrocracks and weak structures which already occurred water-ice phase transition, so that the frost heaving damage and failure in these parts will be aggravated.

## Conclusion

1. The coal-rock stress-strain curve subject to the low temperature-impact load coupling effect has the following features: The linear elastic segment with relatively large slope, compared to the room temperature, the proportion of the linear elastic segment in the whole curve increased and the slope sharply increased, which indicates that the initial modulus of the coal-rock at low temperature increased and its anti-deformation capacity enhanced; the longer elastic deformation segment, during which the elastic modulus significantly decreased, the stress steadily increased with the strain, this curve was closer to a linear elastic change, with obvious brittle characteristics, the internal microcracks initiated and a few propagated stably, the linear elastic deformation predominated the coal-rock deformation; the shorter crack propagation segment, a lot of internal microcracks initiated and transited from stable propagation to unstable propagation, the primary cracks quickly grew to cut through the coal specimen, until a micro failure happened on the specimen; the quick unloading segment, the stress-strain curve quickly decreased at a certain rate after experiencing a transient course of strain softening, which indicates the stronger coal-rock brittle performance.
2. At high strain rate, in 25 °C~−40 °C, the peak stress of the saturated specimen tended to increase then decrease as the temperature fell, the temperature decreased from 25 °C to −5 °C, and the peak strength changed from 32.83 MPa to 34.16 MPa, up by 4%; in −5 °C~−30 °C, the peak strength tended to increase as the temperature fell, which was 37.72 MPa, 38.37 MPa, 40.34 MPa, 30.53 MPa at −10 °C, −15 °C, −20 °C and −30 °C respectively, up by 14.9%, 16.9%, 22.9%, 24.0% respectively if compared to −5 °C; when the temperature fell from −30 °C to −40 °C, the peak strength of saturated specimens decreased significantly to 28.74 MPa from 40.70 MPa at −30 °C, down by approximately 42.2%; in 25 °C~−40 °C, the peak strain tended to decrease and increase, and the turning point of temperature was −10 °C.
3. In 0 °C~−5 °C, although the water-ice phase transition had a little effect on the static load strength, it may deteriorate the coal-rock dynamic strength subject to the strong impact load.
4. The coal-rock dynamic mechanical properties may be deteriorated in −15 °C~−30 °C, because the medium matrix and the ice would shrink as the temperature fell, and the shrinkage rate of the rock matrix was significantly greater than that of the ice medium. Furthermore, since the coal-rock is composed of multiple minerals, the shrinkage rate of mineral particles at low temperature may vary from one to another. Due to the sharp differences in shrinkage rate of multiple media which comprise the frozen coal specimen, cracks, defects and weak structures may initiate on the structural plane of coal specimen and at the interface between the matrix and the ice, and the mechanical properties will be further deteriorated.
